# Advances in computer vision and deep learning-facilitated early detection of melanoma

**DOI:** 10.1093/bfgp/elaf002

**Published:** 2025-03-26

**Authors:** Yantong Liu, Chuang Li, Feifei Li, Rubin Lin, Dongdong Zhang, Yifan Lian

**Affiliations:** Department of Gastroenterology, Zhongshan Hospital of Xiamen University, School of Medicine, Xiamen University, 201 Hubin South Road, Siming district, Xiamen 361005, China; Department of Computer and Information Engineering, Kunsan National University, 558 Daehak Road, miryong district, Gunsan 54150, Republic of Korea; Department of Biological Sciences, Purdue University, 610 Purdue Mall, West Lafayette, IN 47906, United States; Department of Biochemistry and molecular biology, Mayo clinic, MN 55905, United States; Department of Orthopedics, Shenzhen Children's Hospital, 7019 Yitian Road, Futian District, Shenzhen, 518000, China; Department of Gastroenterology, Zhongshan Hospital of Xiamen University, School of Medicine, Xiamen University, 201 Hubin South Road, Siming district, Xiamen 361005, China; Department of Gastroenterology, Zhongshan Hospital of Xiamen University, School of Medicine, Xiamen University, 201 Hubin South Road, Siming district, Xiamen 361005, China

**Keywords:** object detection, image segmentation, edge detection, computer vision, melanoma, skin cancer

## Abstract

Melanoma is characterized by its rapid progression and high mortality rates, making early and accurate detection essential for improving patient outcomes. This paper presents a comprehensive review of significant advancements in early melanoma detection, with a focus on integrating computer vision and deep learning techniques. This study investigates cutting-edge neural networks such as YOLO, GAN, Mask R-CNN, ResNet, and DenseNet to explore their application in enhancing early melanoma detection and diagnosis. These models were critically evaluated for their capacity to enhance dermatological imaging and diagnostic accuracy, crucial for effective melanoma treatment. Our research demonstrates that these AI technologies refine image analysis and feature extraction, and enhance processing capabilities in various clinical settings. Additionally, we emphasize the importance of comprehensive dermatological datasets such as PH2, ISIC, DERMQUEST, and MED-NODE, which are crucial for training and validating these sophisticated models. Integrating these datasets ensures that the AI systems are robust, versatile, and perform well under diverse conditions. The results of this study suggest that the integration of AI into melanoma detection marks a significant advancement in the field of medical diagnostics and is expected to have the potential to improve patient outcomes through more accurate and earlier detection methods. Future research should focus on enhancing these technologies further, integrating multimodal data, and improving AI decision interpretability to facilitate clinical adoption, thus transforming melanoma diagnostics into a more precise, personalized, and preventive healthcare service.

## Introduction

Dermatological research increasingly focuses on the early detection of melanoma, which is crucial for improving patient survival rates. Significant progress in monitoring and evaluating nevi suggestive of melanoma has been catalyzed by leveraging cutting-edge AI, especially through computer vision and deep learning [1, 2]. The integration of AI methodologies with traditional dermatoscopic analysis has created a sophisticated classification pipeline that enhances diagnostic precision by combining deep learning-driven image analysis with hand-crafted features [3].

Due to the lack of effective diagnostic methods for melanoma in its early stages, it is often challenging for individuals to determine whether they have melanoma without seeking specialized care. Early detection of melanoma can significantly enhance the likelihood of a positive outcome, with a more favorable prognosis for those diagnosed at an early stage. A thorough examination of these methodologies in the literature reveals substantial avenues for optimizing and refining AI frameworks aimed at the early diagnosis of skin cancers [4, 5]. The integration of color metrics derived from dermoscopy images that are useful for clinical assessment underscores the importance of multifaceted image analysis in achieving robust diagnostic results [6]. The integration of color metrics derived from dermoscopy images that are useful for clinical assessment underscores the importance of multifaceted image analysis in achieving robust diagnostic results, indicating the feasibility of incorporating diverse imaging modalities to improve early melanoma detection [7]. The use of computer vision systems in clinical settings, capable of providing diagnostic results with varying levels of confidence based on the extraction of specific dermatoscopic features, has shown potential to improve the reliability and accuracy of melanoma diagnosis [8]. The development of a web-based application featuring a deep convolutional neural network marks significant progress by enabling remote melanoma identification, thereby enhancing the accessibility and utility of this technology. Despite these advancements, it is important to note that some detection algorithms, especially those relying on standard RGB camera photography, still do not match the diagnostic precision of skilled dermatologists. This finding prompts critical examination of the effectiveness of emerging *versus* traditional diagnostic methods [5]. The collective exploration of deep learning, broader AI applications, and advanced imaging technologies has profoundly transformed melanoma detection, promising significant improvements in clinical practices and patient care outcomes. Nevertheless, continuous refinement and clinical integration of these technologies are crucial to fully realize their potential to revolutionize melanoma diagnosis and treatment.

Artificial intelligence applications can enhance skin cancer screening in primary care settings and streamline dermatologist referrals. Referral data from primary care physicians (PCPs) to teledermatology consultations were used to train a model with a top 3 accuracy and specificity of 93% and 83%, respectively [9]. This performance was comparable to dermatologists and exceeded that of PCPs and nurse practitioners. It is also important to compare the diagnostic accuracy of different models trained on different datasets in order to accurately assess their potential for clinical application and to help make a contribution to early diagnosis and their prognostic recovery [10].

Recent advances in AI-assisted melanoma detection provide promising solutions for addressing the challenges of this aggressive cancer [11]. Historically, melanoma’s early detection has been challenging due to the subjective nature and variability of traditional diagnostic methods, such as dermatologists’ visual assessment of dermoscopic images [12]. This variability has driven the development of automated computer vision technologies, aimed at reducing subjectivity, improving diagnostic accuracy, and streamlining the diagnostic process [1, 6].

Notably, the study by Alwakid *et al.* [1] utilizing the HAM10000 dataset exemplifies the implementation of deep learning strategies in dermatological diagnostics, highlighting the potential of these technologies to refine and augment traditional methods. Additionally, Shorfuzzuzzaman’s [2] development of an explainable stacked ensemble of deep learning models has been instrumental in improving the interpretability of AI-driven decisions, thus aiding dermatologists in clinical decision-making. These technological advancements align with the pressing need to mitigate the limitations of conventional diagnostics. The research by Sankarapandian *et al.* [4], demonstrating the effectiveness of a Pathology Deep Learning System (PDLS) in the triage of melanoma specimens, underscores the potential of AI to alleviate the burden on dermatopathologists by streamlining specimen sorting and triage. Furthermore, investigations into the efficacy of diverse diagnostic approaches by Saba [6] and the innovative use of sparse auto-encoders and SVMs for melanoma classification by Scharcanski and Jacob [7] have shown remarkable specificity, sensitivity, and accuracy, bolstering the case for AI in dermatology. The collective impact of these technologies suggests a transformative potential for enhancing the precision of melanoma diagnostics, ultimately aiming to improve patient survival and quality of life through more effective early detection and treatment [1, 4]. As these technologies continue to evolve and undergo rigorous validation, their integration into routine clinical practice holds the promise of significantly advancing melanoma diagnostics and therapeutic strategies.

In this review, we address critical gaps in the current melanoma detection methodologies by harnessing the capabilities of artificial intelligence. Artificial intelligence offers transformative potential to improve diagnostic accuracy, reduce variation between clinicians and detect melanoma earlier, which is expected to be instrumental in improving early diagnosis for patients in different regions. Our study uniquely compiles and evaluates a wide array of AI technologies, highlighting their respective strengths and challenges, and proposes a framework for their integration into existing clinical workflows. This synthesis not only underscores the critical role of AI in advancing dermatological diagnostics but also charts a path forward for its broader adoption in clinical settings.

**Figure 1:**
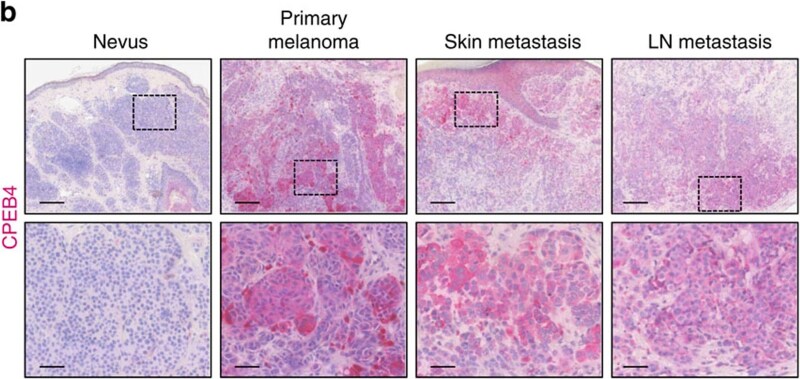
Representative micrographs of sections. Scale bars, 200 μm (upper images); and 50 μm (lower images) [26].

**Figure 2:**
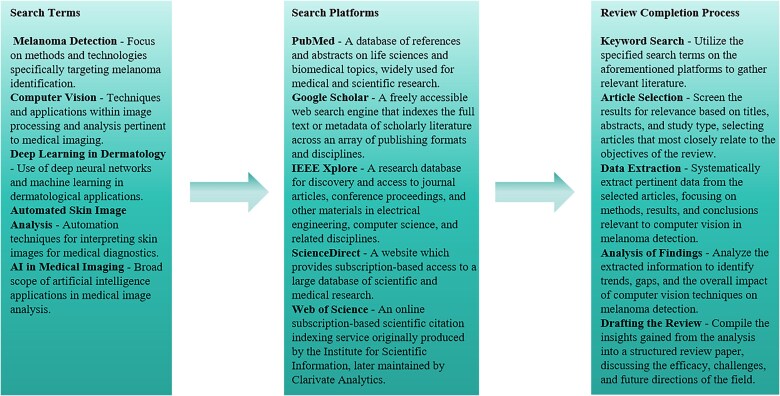
Literature review methodological framework.

The surge of innovations in computer vision and deep learning has notably impacted the domain of early disease detection, with melanoma as a prime focus. The advent of high-performance deep learning frameworks, such as PyTorch, has empowered researchers to deploy complex models efficiently for early detection purposes [13]. The exploration of adversarial robustness in neural networks introduces vital security considerations [14–17]. The reimagining of deep learning architectures to optimize computational efficiency, as seen in the restructuring of the Inception architecture for computer vision, has facilitated the scaling of networks to enhance their performance significantly [18–20]. Moreover, the incorporation of Bayesian methods to model uncertainty in deep learning presents a promising avenue to enhance model reliability [21, 22]. In the realm of medical imaging, deep learning models have become a focal point for advancing melanoma detection. Studies have extensively explored the implementation of Enhanced Super-Resolution Generative Adversarial Networks (ESRGAN) to augment the capabilities of deep learning frameworks in identifying melanoma with greater accuracy [23–25]. Concurrently, the evolution of deep convolutional networks, particularly exemplified by the development of InSiNet, has been pivotal in refining approaches to skin cancer detection and segmentation [25]. Additionally, the application of gray level co-occurrence matrix features combined with sophisticated machine learning techniques has emerged as a potent tool for the effective clinical diagnosis of melanoma [14]. Addressing the broader spectrum of melanoma epidemiology and the imperative of early detection, recent research has specifically highlighted the variability and disparities prevalent in melanoma epidemiology across Europe, emphasizing the necessity for enhanced registration, early diagnosis, and proactive prevention strategies [15]. Figure 1 shows representative micrographs of sections from the indicated lesions showing CPEB4 staining in pink. Nuclei are counterstained with haematoxylin. Figure 2 provides a structured overview of the methodology used in conducting a literature review on computer vision for early melanoma detection. It details the search terms, platforms utilized for literature acquisition, and the sequential steps of the review process. We set a prerequisite before all searches that the search results must contain ‘Melanoma Detection’.

This diagram serves as a concise guide to understanding the comprehensive approach taken in the analysis. Thus, the integration of state-of-the-art computer vision and deep learning technologies has significantly advanced the early detection of melanoma. This synergy of cutting-edge deep learning techniques and medical imaging is poised to revolutionize early detection methodologies, promising to substantially improve patient outcomes by enhancing the accuracy and timeliness of diagnoses [27].

## Existing melanoma datasets

The advancement of melanoma diagnostic methods through artificial intelligence critically depends on the availability and quality of dermatological image datasets. These datasets are essential for training and validating algorithms designed to automate and improve melanoma detection and classification. As AI technologies evolve, diverse and comprehensive datasets become more crucial, forming the empirical foundation for algorithms that potentially exceed human diagnostic capabilities. The synergy between technological advancements and empirical data is reflected in the expanded and enriched datasets tailored for melanoma detection. The following discussion details these key datasets, their unique characteristics, and their impact on AI-assisted melanoma diagnosis. As shown in Table 1 a summary of the current major datasets on melanoma.

**Table 1:** Major datasets on melanoma.

Dataset Name	Availability	Number of Images	Number of Melanomas
PH2	Publicly available	200	40
ISIC 2016	Publicly available	900	273
ISIC 2017	Publicly available	2000	374
ISIC 2018 (HAM10000)	Publicly available	10,015	1113
ISIC 2019	Publicly available	25,333	4522
ISIC 2020	Publicly available	33,126	584
DERMQUEST	Publicly available	126	66
MED-NODE	Publicly available	170	100
DERMNET	Publicly available	22,500	635
DERMIS	Publicly available	397	146
DERMOFIT	Purchase required	1300	76
ISIC 2024	Publicly available	401,059	Over 10,000

**Figure 3:**
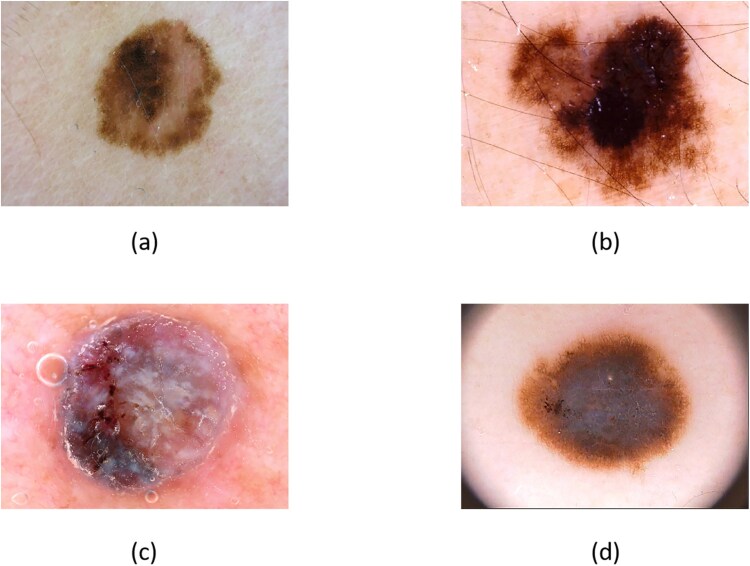
ISIC datasets.four dermoscopic images labeled **(a)**, **(b)**, **(c)**, and **(d)**, each showing different skin lesions to highlight their diverse characteristics in dermatological evaluation.

The field of melanoma research has substantially benefited from various publicly available datasets, which have driven advancements in AI-assisted diagnostic tools. Notably, the PH2 dataset from the Dermatology Service of Hospital Pedro Hispano, Portugal, includes 200 dermoscopic images comprising 40 melanomas and 160 other skin lesions [28]. From 2016 to 2020, the International Skin Imaging Collaboration (ISIC) released datasets progressively, with the ISIC 2020 dataset containing over 33,000 images representing a wide range of skin conditions [29–34]. and The dataset ISIC 2024 of skin abnormalities including melanoma for the Kaggle challenge was officially released on 27 June 2024 [35]. The HAM10000 dataset, significant for its 10,015 images, has been instrumental in enhancing algorithmic training with its diversity of skin lesion presentations [36]. Lesser-known but vital, the DERMQUEST and MED-NODE datasets provide varied image types and diagnostic challenges, enriching the data pool for algorithm training and testing [37, 38]. These datasets vary not only in size, ranging from hundreds to tens of thousands of images, but also in the depth of annotations and the diversity of lesion types included. For instance, DERMNET hosts 60 000 images, one of the largest collections, whereas DERMIS and DERMOFIT feature detailed annotations crucial for training sophisticated machine learning models [39–41]. The availability of these datasets enables the training, testing, and validation of diverse diagnostic algorithms, including convolutional neural networks (CNNs) and more complex hybrid models incorporating techniques such as support vector machines (SVM) and decision trees [42, 43]. This robust dataset infrastructure enhances algorithmic accuracy and specificity and supports the exploration of model efficiency and optimization using techniques like ADAM and stochastic gradient descent [44]. The collective contribution of these datasets forms the cornerstone of improved melanoma detection, providing data to underpin the development of advanced diagnostic tools, leading to the development of models that are more appropriate for different populations and are expected to contribute to the early detection of patients. Figure 3 shows part of the image dataset for the ISIC public melanoma identification challenge. Table 2 shows the URLs of the links to the main datasets for melanoma, making it easier to find them.

**Table 2:** Links to major datasets on melanoma URL.

Dataset Name	URL (accessed on 30 May 2024)
PH2	https://www.fc.up.pt/addi/ph2%20database.html
ISIC 2016	https://challenge.isic-archive.com/landing/2016/
ISIC 2017	https://challenge.isic-archive.com/landing/2017/
ISIC 2018	https://dataverse.harvard.edu/dataset.xhtml?persistentId=doi:10.7910/DVN/DBW86T
(HAM10000)	
ISIC 2019	https://challenge.isic-archive.com/landing/2019/
ISIC 2020	https://challenge2020.isic-archive.com/
DERMQUEST	https://www.kaggle.com/datasets/fatemehmehrparvar/skin-cancer-detection
MED-NODE	https://paperswithcode.com/dataset/med-node
DERMNET	https://dermnetnz.org/dermatology-image-dataset
DERMIS	https://www.dermis.net/dermisroot/en/home/index.htm
DERMOFIT	https://licensing.edinburgh-innovations.ed.ac.uk/product/dermofit-image-library
ISIC 2024	https://challenge2024.isic-archive.com/

## Deep learning for melanoma detection

Deep learning has transformed melanoma detection. Neural networks, especially deep CNNs and transfer learning (TL), have become pivotal in advancing skin lesion detection, segmentation, and classification. Historically, enhancing system performance involved designing deep networks with multiple hidden layers—both convolutional and fully connected (FC)—to achieve superior statistical outcomes, crucial for clinical diagnostics. Although this design strategy initially increased computational demands, the primary focus was on achieving higher accuracy and reliability, essential for medical applications.

From 2018 to 2024, deep learning in melanoma detection primarily used architectures like ResNet and VGG, known for effectively handling and interpreting complex dermoscopic imagery. According to an analysis of the Web of Science database, these deep networks, capable of forming hierarchical feature representations, were consistently preferred. According to an analysis of the Web of Science database, these deep networks, capable of forming hierarchical feature representations, were consistently preferred. These networks evolved during this period, maintaining their popularity beyond 2021.

Newer architectures like YOLO (You Only Look Once), from YOLOv3 to YOLOv8, were adapted for real-time, accurate skin lesion detection. These models enable rapid lesion localization and enhanced feature extraction, crucial for distinguishing between benign and malignant lesions. Additionally, innovations like attention mechanisms and capsule networks have been explored to enhance model specificity and sensitivity. These advances have significantly expanded the capabilities of AI-assisted melanoma diagnostics, improving both the efficiency and accuracy of detection tools and providing dermatologists with more reliable support in clinical settings.

As the field advanced towards 2024, developments in deep learning continued to refine models, reducing computational overhead while maintaining or improving diagnostic accuracy. Innovations like lightweight network architectures, enhanced TL techniques, and novel training methodologies such as federated learning have become prominent. The adoption of explainable AI principles in melanoma detection has gained traction, aiming to enhance the transparency and comprehensibility of neural network decision-making for dermatologists. This shift is crucial for increasing clinical acceptance of AI tools, as it offers insights into the diagnostic pathways and decision rationales of the models. The technical route is shown in Table 3.

**Table 3:** Statistical table of algorithms used for melanoma diagnosis.

**Algorithms**	**Technical direction**	**Description**
ResNet [23, 39, 45–56](2015)	ResNet 34, ResNet 50, SEResNet 50, ResNet 101, ResNet 152, FCRN	Residual Network, leverages a unique architecture that allows certain layers to skip over others, making it possible to build much deeper networks.
GAN [57–61](2014)	GAN, SPGGAN, DCGAN, DDGAN, LAPGAN, PGAN, Conditional GAN	Employs generative adversarial networks to create synthetic images of skin lesions, aiding in training dataset augmentation.
Xception [61–66](2016)	Xception, XceptionV2	Adopts depthwise separable convolutions to provide a more efficient structure than standard convolutions, suitable for high-resolution images.
DenseNet [67–71](2017)	DenseNet 121, DenseNet 161, DenseNet 169, DenseNet 201, DenseNet 264	Features densely connected convolutional networks that ensure maximum information flow between layers, enhancing feature retention.
VGG [44, 71–75](2014)	VGG 16, VGG 19, VGG 21	Notable for its straightforward architecture, consisting primarily of stacked convolutional layers. This design allows the network to deeply process images through multiple filters, capturing intricate details, which is vital for tasks like image recognition.
ResNeXt [76](2017)	ResNeXt, SeResNeXt, SeResNeXtDense	ResNeXt extends the principles of ResNet by incorporating grouped convolutions, which divide the input into smaller chunks processed by different filters.
Inception/GoogLeNet [77] (2014)	GoogLeNet (Inception v2), InceptionResNet-v2, Inception v3, Inception v4, Inception v5	Incorporates modules with parallel convolutions of different sizes, improving the network’s ability to recognize patterns at various scales.
NASNet [78, 79](2017)	NASNet, NASNet-Large, NASNet Mobile	Uses neural architecture search to optimize its structure, enhancing its efficiency and accuracy in image classification tasks.
U-Net [80–84](2015)	U-Net, U-Net++	A convolutional network for precise segmentation, crucial for delineating the boundaries of melanoma in dermoscopic images.
AlexNet [85–88](2012)	AlexNet, AlexNet Modified	Early deep learning network that popularized the use of CNNs in image recognition, still used for baseline comparisons.
MobileNet [89, 90](2017)	MobileNet, MobileNetV2, MobileNetV3	Designed for mobile and edge devices, these networks provide a balance between speed and accuracy, useful for real-time applications.
YOLO [91–95](2016)	YOLO v3, YOLO v4, YOLO v5, YOLO v6, YOLO v7, YOLO v8, YOLOX	Real-time object detection system that has been progressively improved to provide faster detection with higher accuracy.
Mask R-CNN [96–100] (2017)	Mask R_CNN, Mask R_CNN with FPN	Extends Faster R-CNN by adding a branch for predicting segmentation masks on each Region of Interest (RoI), effective for detailed lesion segmentation.
Random Forest [101] (1990)	**/**	A traditional machine learning algorithm that uses ensemble learning techniques for classification, often used in conjunction with other methods for feature analysis.
SVM [24](1990)	Linear SVM, Kernel SVM	Support Vector Machine, a robust classifier frequently used in medical image analysis due to its effectiveness in high-dimensional spaces.
EfficientNet [102–105] (2019)	EfficientNet, EfficientNetB5, EfficientNetB6, EfficientNetB7	Optimizes scaling for width, depth, and resolution of networks, providing a balanced architecture for various computational budgets.

This review extends and differs from previous work. For example, the review by Harsh Bhatt *et al.* [106] focuses extensively on various machine learning algorithms such as k-nearest neighbors, SVM, and CNNs for skin cancer detection, which are specific to machine learning only and have a much broader scope of skin cancer. Our manuscript, on the other hand, focuses more on the current state of the art in melanoma, which includes machine learning and deep learning, explores the methods more broadly, and examines specific applications and advances in melanoma early detection.

**Figure 4:**
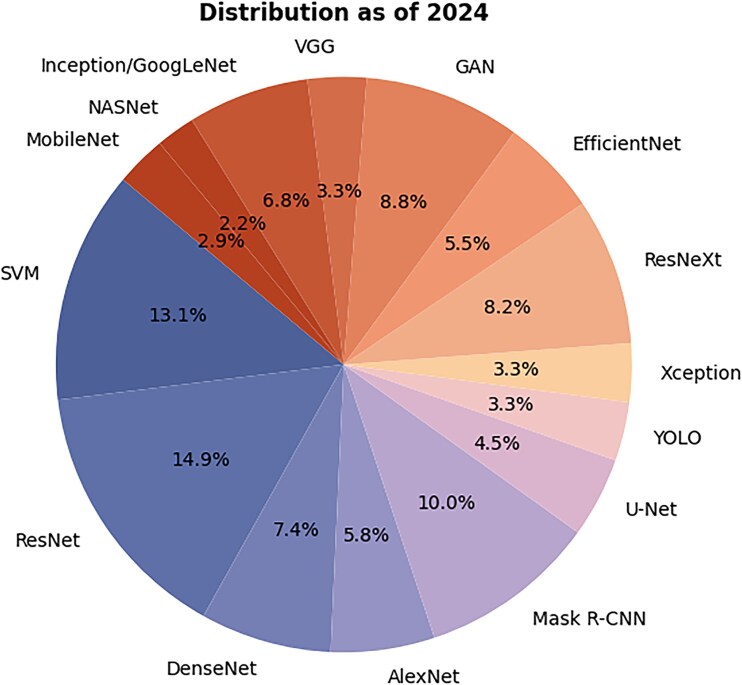
Classification of technical means as of 2024.

Figure 4 shows the proportional use of various machine learning and deep learning models as of 2024, depicted in a detailed pie chart. The chart reveals that ResNet, the most commonly used model, accounts for 14.9% of applications, followed closely by SVM with 13.1%. Other significant models are DenseNet and Mask R-CNN, representing 10.0% and 8.2% of total usage, respectively. Additionally, models such as EfficientNet and GAN constitute a smaller, yet notable, share of the distribution. This chart effectively showcases the field’s diversity and specialization, highlighting both traditional models such as AlexNet and newer, specialized technologies like Mask R-CNN.

Despite the surge in the application of advanced neural network models, SVM remains a popular choice for melanoma detection due to its robustness and effectiveness in high-dimensional spaces, which are common in image-based data. SVM’s enduring popularity stems from its ability to find the optimal boundary between classes while minimizing generalization error, which is critical for medical diagnostics. Compared to newer Neural Network (NN) models, SVMs can be more interpretable and less resource-intensive, making them suitable for scenarios where computational resources are limited or where model transparency is paramount. In addition, SVMs often require less data to train effectively, which is beneficial in medical fields where annotated data can be scarce.

The integration of multimodal data, combining dermoscopic images with clinical patient data and genetic markers, is being explored to enhance the predictive accuracy and personalization of melanoma diagnostics. The trajectory of deep learning in melanoma detection features continuous refinement of technologies and methodologies, driven by the goals of maximizing diagnostic performance and ensuring practical applicability in real-world settings.

We aim to explore computational techniques revolutionizing melanoma detection, acknowledging space constraints and the multitude of available models. In addition, we consider some of the details characterising traditional papers [107–109].

In the realm of melanoma detection, deep learning models have been pivotal in enhancing diagnostic accuracy and efficiency. For instance, CNNs have been extensively applied to analyze dermatoscopic images. The DenseNet architecture, specifically, has shown significant promise due to its ability to process complex image data and retain critical information through its densely connected layers. This model was used in a study where it outperformed traditional models in identifying malignant melanoma lesions, achieving a high accuracy rate [67–71]. Similarly, the YOLO model has been adapted for real-time melanoma detection, enabling the identification and localization of skin lesions rapidly and with notable precision [91–95]. These models exemplify the advanced capabilities of AI in recognizing subtle patterns that are often missed in manual examinations.

Therefore, we have selected a focused set of classic and widely applicable models for detailed discussion. Our selection criteria include proven effectiveness, widespread adoption, and ability to illustrate key principles of modern medical image analysis. Each selected model provides unique insights into dermatological diagnostic challenges and solutions, offering a comprehensive view of current state-of-the-art technologies. Subsequent sections will explore each model’s architecture, functionality, and role in enhancing melanoma detection.

### YOLO for melanoma detection

**Figure 5:**
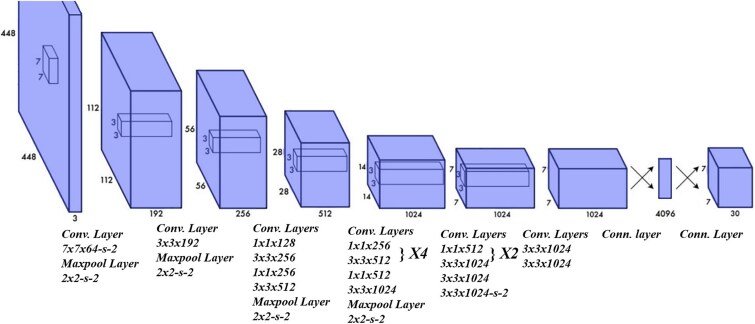
YOLO Neural Network Architecture [110].

In the field of dermatological imaging, the YOLO architecture offers a robust framework for rapid and effective melanoma detection. This approach is particularly advantageous due to its ability to process images in real-time while maintaining high accuracy. Figure 5 illustrates the detailed structure of the YOLO network employed for this purpose. It begins with an input layer that handles 448x448 pixel images, followed by a sequence of convolutional and maxpool layers. The initial convolutional layer applies a 7×7 filter, succeeded by a 2×2 maxpool layer, which reduces the spatial dimension while increasing the depth of feature maps. Subsequent layers alternate between convolutions and maxpooling, with filter sizes and layer depths varying to capture a comprehensive range of features at different scales. The architecture culminates in several FC layers that consolidate these features into final outputs, determining the presence and characteristics of melanomas with remarkable precision. This structured layer progression allows the YOLO model to effectively identify and classify melanomas based on visual data, making it a critical tool in modern medical diagnostics.

### GAN for melanoma detection

**Figure 6:**
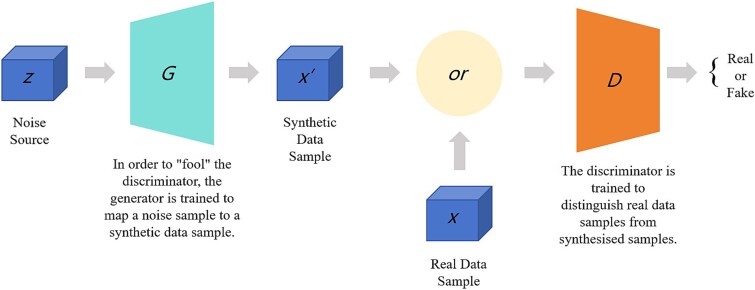
Generative Adversarial Network (GAN) Setup [111].

In the quest to improve melanoma detection using Generative Adversarial Networks (GANs), dynamic interactions between generative and discriminative models are often utilized to improve diagnostic accuracy. Figure 6 depicts a GAN architecture in which the generator (G) creates synthetic images from latent space, aiming to replicate actual dermatoscopic skin lesion samples. The discriminator (D) then evaluates these synthetic images alongside real samples to assess their authenticity. This iterative process allows the D to refine its ability to differentiate between real and synthetic images, leading to progressively more realistic images. This technique enhances both the diversity of training data and the robustness of melanoma detection models, making GANs a valuable tool in medical image analysis.

In the latest field of GANs for melanoma detection, recent studies have provided substantial evidence of their effectiveness. Zhao *et al.* [57] demonstrated the application of StyleGAN combined with DenseNet201 for dermoscopy image classification, achieving enhanced diagnostic accuracy. Similarly, Gong *et al.* [58] utilized StyleGANs with decision fusion techniques to improve classification reliability. Further advancements by Gu *et al.* [59] in progressive TL and adversarial domain adaptation highlight the potential for GANs to generalize across different domains, enhancing their utility in diverse clinical environments. Baur *et al.* [60] introduced MelanoGANs, which specialize in high-resolution skin lesion synthesis, providing a novel method for generating detailed training data. Lastly, Yi *et al.* [61] explored unsupervised and semi-supervised learning approaches using categorical GANs, assisted by Wasserstein distance, to categorize dermoscopy images with minimal labeled data requirements. These studies collectively underscore the transformative potential of GANs in the field of dermatological imaging, presenting a robust framework for both enhancing data quality and improving diagnostic models.

### Mask R-CNN for melanoma detection

**Figure 7:**
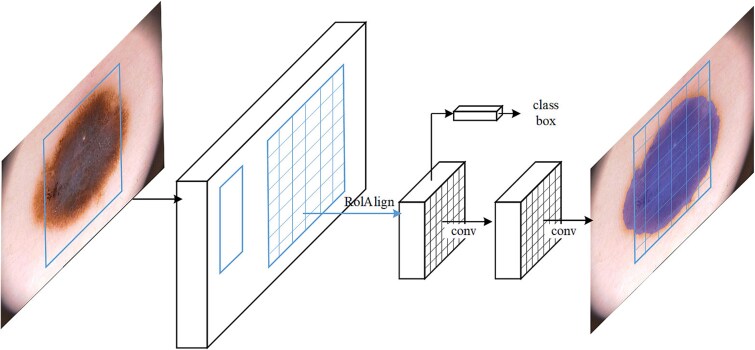
Mask R-CNN [112] Architecture for Precise Melanoma Segmentation.

The use of Mask R-CNN in melanoma detection represents a major advancement in precision diagnostics, facilitating detailed segmentation and classification of skin lesions. This method employs deep learning to identify and analyze complex features in dermoscopic images, improving melanoma detection accuracy. Figure 7 depicts the Mask R-CNN architecture, starting with a backbone network that extracts feature maps from the input images. A Region Proposal Network processes these features to identify regions of interest. Subsequent FC and convolutional layers refine these proposals, classify them, and perform box regression for precise localization. Finally, the network applies segmentation masks to the detected regions, precisely delineating melanomas from healthy skin and enhancing diagnostic accuracy and treatment planning.

### ResNet for melanoma detection

**Figure 8:**
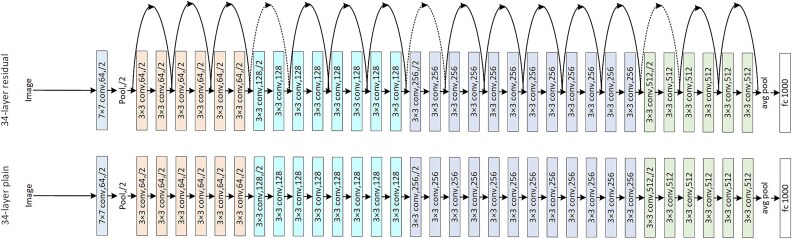
Detailed View of ResNet Layer Configuration.

**Figure 9:**
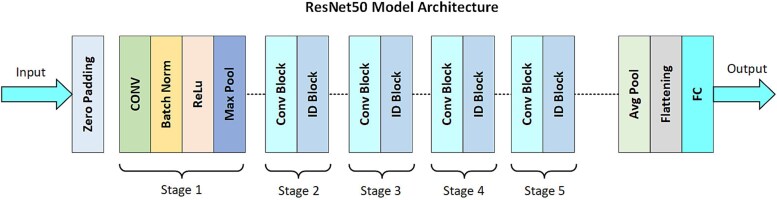
ResNet50 [113] Model Flow.

The integration of ResNet-based architectures represents a significant advancement in developing sophisticated melanoma detection methods. These models enhance deep learning processes, excelling at identifying complex patterns in dermoscopic images—essential for accurate and early melanoma diagnosis. Figures 8 and 9 illustrate the detailed structures of these architectures. Figure 8 depicts a hypothetical Resknet model with interconnected layers, showing a sequence of transformations from initial inputs through convolutional stages, each defined by specific filter sizes and stride details. Figure 9 presents the ResNet50 model architecture, a prominent configuration for image processing, featuring convolutional layers, batch normalization, ReLU activation, and pooling, culminating in output *via* average pooling and FC layers. Both figures underscore the systematic layering and functionality essential for processing and analyzing medical imaging data, thereby improving melanoma detection and classification.

### Densen for melanoma detection

**Figure 10:**
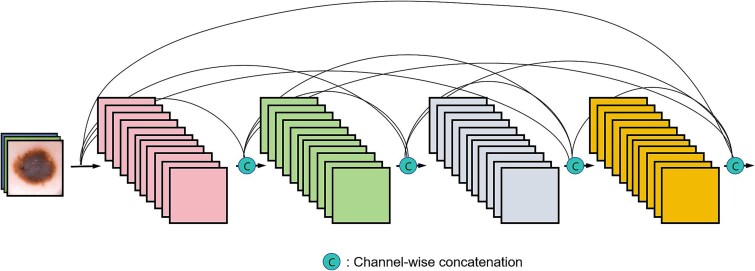
DenseNet [114] Connectivity Diagram.

The use of DenseNet architectures has greatly influenced medical imaging, especially in accurately detecting and analyzing melanoma. Designed to optimize information flow, these networks ensure each layer receives inputs from all previous layers, enhancing the model’s learning capabilities. Figure 10 displays a typical DenseNet architecture used in melanoma detection. It illustrates how each layer progressively receives and merges feature maps from all previous layers through depth concatenation. This approach enables the network to be both deep and efficient, capturing complex features essential for detecting subtle nuances in melanoma images. The diagram emphasizes the model’s depth and sophistication, demonstrating how such architectures enhance diagnostic accuracy in dermatology.

## Evaluation criteria

In our analysis of melanoma detection models, we discuss standardized statistical metrics commonly used to assess their accuracy and efficacy, providing a theoretical framework for their evaluation in clinical studies. Accuracy, precision, recall, and the F1 score are crucial for assessing these models, offering a comprehensive view of their performance in different scenarios. Calculated using specific formulas, these metrics act as critical benchmarks, allowing us to systematically quantify each model’s effectiveness. This systematic approach enables algorithm refinement, improving their accuracy in melanoma identification and minimizing false positives and negatives—a key aspect of medical diagnostics. These theoretical underpinnings and metrics are commonly used measures in the field, and by incorporating these metrics into the corresponding testing phases of the individual models, the clinical accuracy of the models can be greatly improved, providing healthcare professionals with reliable support for early melanoma diagnosis, and thus saving lives, the.


(1) \begin{equation*} \mathrm{P}=\frac{\mathrm{TP}}{\mathrm{TP}+\mathrm{FP}} \end{equation*}

(2) \begin{equation*} \mathrm{R}=\frac{\mathrm{TP}}{\mathrm{TP}+\mathrm{FN}} \end{equation*}

(3) \begin{equation*} \mathrm{AP}=\frac{\mathrm{TP}+\mathrm{TN}}{\mathrm{TP}+\mathrm{FN}+\mathrm{FP}} \end{equation*}

(4) \begin{equation*} \mathrm{mAP}=\frac{1}{\mathrm{Q}}\sum_{\mathrm{q}=\mathrm{Q}}\mathrm{AP}\left(\mathrm{q}\right) \end{equation*}

Daniel Kvak developed a melanoma detection algorithm based on the CoAtNet architecture, a hybrid model that combines CNNs and Transformer models. The model was evaluated using precision, recall, and average precision, achieving high values: 0.901, 0.895, and 0.923, respectively, demonstrating robust performance in melanoma detection [115]. H. Iyatomi *et al.* applied a computer-based method to extract melanoma tumors from dermoscopy images using precision and recall as evaluation metrics. Their refined algorithm achieved a precision of 94.1% and recall of 95.3%, proving its effectiveness in mimicking dermatologists’ performance [116]. Manar Elshahawy *et al.* introduced a hybrid YOLOv5 and ResNet-based technique for melanoma detection, evaluated with precision, recall, and mAP. Their model achieved exceptional performance, with precision at 99.0%, recall at 98.6%, and mAP between 0.0 and 0.95 reaching 98.7%, setting a high benchmark for melanoma detection algorithms [117].

## Conclusion

Our study underscores the crucial role of diverse and comprehensive datasets such as PH2, ISIC, DERMQUEST, and MED-NODE, which supply vital data for algorithm training and testing. This ensures robustness, versatility, and performance under diverse clinical conditions.

In conclusion, continuous advancements in AI and machine learning are redefining medical imaging standards, particularly in melanoma detection. As these technologies advance, they promise enhanced diagnostic accuracy, efficiency, and accessibility, significantly impacting public health. Effective integration of these technologies into clinical practice necessitates ongoing collaboration among technologists, clinicians, and researchers to maximize and effectively implement AI benefits in practical settings.

The integration of AI into melanoma detection extends beyond theoretical applications, demonstrating substantial benefits in clinical settings. For instance, CNNs have been deployed in dermatological clinics to analyze dermoscopic images, significantly enhancing the precision of early-stage melanoma identification. Studies have shown that these AI-enhanced diagnostics reduce the rate of false negatives, a common challenge in traditional visual inspections by dermatologists. Furthermore, the use of GANs in creating detailed training datasets has improved the robustness of these AI systems, leading to more reliable diagnoses. The practical application of these technologies not only promises to elevate diagnostic accuracy but also improves patient outcomes by facilitating early and tailored treatments, thereby potentially increasing survival rates and reducing treatment costs. Future research will focus on refining these AI models and expanding their application to ensure that they can be seamlessly integrated into routine clinical practice, further transforming the landscape of melanoma detection and treatment.

Future research should focus on refining these technologies, integrating multimodal data, and enhancing AI decision explainability to bridge the gap between technology capabilities and clinical needs. This will lead to more personalized, precise, and preventive healthcare solutions, establishing AI as an indispensable tool in combating melanoma.

**Highlights Key points**

- The paper conducts a comprehensive review of advanced AI models such as CNN, YOLO, GAN, Mask R-CNN, ResNet, and DenseNet, highlighting their pivotal role in enhancing early melanoma detection through the latest datasets. It categorizes these models based on their potential for integration into clinical workflows.
- Analyzes the significant challenges in diagnosing early melanoma, particularly in settings lacking specialized care, underscoring the necessity for more accessible diagnostic tools.
- Discusses the transformative advances in AI technologies, which have led to more reliable and accurate melanoma diagnoses in clinical environments, marking a significant step forward in dermatological care.
- Presents a meticulously organized and comprehensive publicly available melanoma dataset, complete with download addresses, serving as a valuable resource for researchers and clinicians alike.
- Provides an introductory overview of various high-performing AI models and offers a comparative analysis, shedding light on their respective strengths and applications in the field of melanoma detection.
- Explores the ongoing development and future trajectories of AI-assisted melanoma detection, emphasizing the integration of multimodal data and the enhancement of model interpretability to broaden clinical adoption and improve patient outcomes.

**Summary Key points**

- **Advanced AI Techniques:** The paper reviews the application of advanced artificial intelligence models like CNN, YOLO, GAN, Mask R-CNN, ResNet, and DenseNet in enhancing early melanoma detection, emphasizing their ability to improve diagnostic accuracy and speed.
- **Challenges in Clinical Integration:** It highlights the challenges that automated computer vision technologies face in clinical settings, such as data bias and the need for extensive data annotation, which can impact the generalizability and effectiveness of these models.
- **Importance of Diverse Datasets:** The study underscores the critical role of diverse and comprehensive datasets like ISIC Archive and HAM10000 in training AI models, ensuring robustness and versatility in their diagnostic capabilities.
- **Future Directions:** The paper discusses ongoing developments and future directions in AI-assisted melanoma detection, including the integration of multimodal data and enhancing model explainability to facilitate wider clinical adoption.
- **Impact on Patient Outcomes:** It suggests that with continued refinement and validation, AI technologies are poised to significantly enhance early melanoma detection and treatment, potentially improving patient survival rates and quality of life.

## Data Availability

The datasets generated and analyzed during the current study are available from the corresponding author upon reasonable request.

## Notes

**Yantong Liu:** (Member, IEEE) received PhD in Computer Science from Kunsan National University and worked as a researcher. He also serves as an off-campus research consultant tutor at Zhongshan Hospital of Xiamen University. His research interests include AI medicine, Computer Vision, LLM applications, and other AI interdisciplinary subjects.

**Chuang Li:** Chuang is a Senior Scientist at Department of Research and development, Abbvie Inc, US. He earned his Ph. D. degree in Biological Science from Purdue University, US. Chuang’s research interests focus on the application of Artificial Intelligence across interdisciplinary fields, including Modern agriculture, Disease early diagnosis, Next generation medicine.

**Feifei Li:** Currently a PhD student at Mayo Clinic, main research areas: cancer attack, neural networks, smart medicine

**Rubing Lin**: Graduated from Shenzhen Hospital of Peking University, and is now a doctor at Beijing Children’s Hospital, with research areas: oncology, health management.

**Dongdong Zhang:** PhD student at Zhongshan Hospital of Xiamen University, received project funding from National Natural Science Foundation of China and served as a participant. Main research areas: AI medical, cancer, gastrointestinal.

**Yifan Lian:** He is a researcher and chief physician at Zhongshan Hospital of Xiamen University, where he received a grant from the National Natural Science Foundation of China and acted as a moderator. Main research areas: AI medicine, cancer, gastrointestinal.
